# Three Reasons Why Expecting ‘Recovery’ in the Context of the Mental Health Impacts of Climate Change Is Problematic

**DOI:** 10.3390/ijerph20105882

**Published:** 2023-05-19

**Authors:** Jo Longman, Rebecca Patrick, Sarah Bernays, Fiona Charlson

**Affiliations:** 1 University Centre for Rural Health, University of Sydney, 61 Uralba Street, Lismore, NSW 2480, Australia; 2 School of Health and Social Development, Deakin University, Burwood, VIC 3125, Australia; rebecca.patrick@deakin.edu.au; 3 School of Public Health, University of Sydney, Sydney, NSW 2006, Australia; sarah.bernays@sydney.edu.au; 4 Department of Global Health and Development, Faculty of Public Health and Policy, London School of Hygiene and Tropical Medicine, London WC1E 7HT, UK; 5 Queensland Centre for Mental Health Research, Queensland Health, Wacol, QLD 4076, Australia; f.charlson@uq.edu.au; 6 School of Public Health, The University of Queensland, Herston, QLD 4006, Australia; 7 Department of Global Health, Institute for Health Metrics and Evaluation, University of Washington, Seattle, WA 98195, USA

**Keywords:** climate change, mental health, mental health recovery, adaptation, psychological

## Abstract

Global warming is bringing with it continued long-term changes in the climate system. Extreme weather-related events, which are already becoming a daily reality around the world, are predicted to be more intense and frequent in the future. The widespread occurrence of these events and climate change more broadly are being experienced collectively and at scale and do not affect populations evenly. These climate changes have profound impacts on mental health and wellbeing. Existing reactive responses include frequent implied and direct references to the concept of ‘recovery’. This is problematic in three ways: it conceives of extreme weather events as single, one-off occurrences; implies their unexpected nature; and contains an integral assumption of an end point where individuals/communities are ‘recovered’. Models of mental health and wellbeing support (including funding) need to change, shifting away from ‘recovery’ towards a focus on adaptation. We argue that this presents a more constructive approach that may be used to collectively support communities.

## 1. Introduction

The most recent IPCC report states that “human-induced climate change, including more frequent and intense extreme events, has caused widespread adverse impacts and related losses and damages to nature and people, beyond natural climate variability”. It also notes that climate change impacts are becoming increasingly complex, and that multiple climate hazards will occur simultaneously [1]. 

More intense, more frequent, and overlapping extreme-weather-related events are already being experienced globally. In Australia, for example, over the last five years, the Far North Coast of New South Wales has experienced floods, drought, fires and then more flooding in addition to the COVID pandemic. Similarly, parts of California in the USA were subjected to vast and intense wildfires in July 2018 and August 2020 and again in July 2021. These events are therefore no longer singular, rare occurrences. They are also experienced collectively and at scale, where huge swathes of the population are directly and indirectly impacted, often in a way which exacerbates existing inequalities, as populations who are socio-economically disadvantaged are disproportionately affected [2].

Climate change has far-reaching impacts on all aspects of human health, perhaps no more so than on mental health [3], where it has demonstrated clear direct and indirect impacts on numerous mental health outcomes ranging from psychological distress to psychiatric-related hospitalisations and even death [3], effects which are predicted to become more acute. The causal pathways through which climate change impacts on mental health are highly complex and operate on a system-wide level, making mental health a truly cross-cutting issue when considering climate risks and the approaches taken to address them [4].

The compound nature of extreme-weather-related events, the collective experience of these events and the huge scale on which they are happening suggest that we need to carefully scrutinize how the impacts of climate change on mental health and wellbeing and our responses to them are perceived.

## 2. Post-Disaster Responses and the Concept of ‘Recovery’

‘Recovery’ is a term frequently used in the context of climate change, disaster and public health emergency literature; however, there is a paucity of studies that evaluate recovery intervention effectiveness and minimal literature that has explored whether recovery approaches for mental health and wellbeing remain apposite as the threats of climate change are realised.

A longstanding approach to managing the corollaries of climate change, such as extreme-weather-related disasters (fires, floods, droughts, etc.), has been a reactive response with the goal being a ‘recovery’ to pre-disaster conditions, often without specificity regarding who or what recovers. For example, in the Sendai Framework, resilience is defined as: “The ability of a system, community or society exposed to hazards to resist, absorb, accommodate to and recover from the effects of a hazard in a timely and efficient manner” [5] (p. 9). Similarly, in Australia, emergency management is “…built on the concept of prevention, preparedness, response and recovery (PPRR)” [6] (p. 3). This is particularly the case for the management of mental health, which has traditionally had a strong focus on post-disaster recovery, with disaster funding becoming available after an event to provide targeted and often short-term mental health support to an affected population.

## 3. The Problem with ‘Recovery’ in the Face of Climate Change

Embedded in this narrative of recovery is an orientation towards extreme-weather-related events as (1) single events (e.g., one-in-one-hundred-year events) and (2) unexpected events and (3) the notion that ‘recovery’ from them is time-limited with a clear end point. We contend that these three interlinked characterisations do not apply to the climate crisis we currently find ourselves in.

Firstly, the focus on each extreme-weather-related event as a single, one-off occurrence diverts attention away from these events as symptoms of our changing climate and, importantly, does not acknowledge or address the compound impacts of multiple events over time. For example, the gradual erosion of landscapes can have profound impacts on mental health and wellbeing, including changes to identity and loss of place. This is particularly the case for communities rooted in the environment as an aspect integral to livelihood [7] and culture [8]. Each year sees greater numbers of communities affected by multiple events [9], and emerging evidence suggests that the health risks of exposure to multiple disastrous events are greater than the risks of single events [10].

Secondly, embedded in this concept of ‘recovery’ from an extreme-weather-related event is an implication of its unexpected nature. Extreme-weather-related events are often responded to and described as though they were a shock and an unexpected and temporary ‘interruption’ to our everyday lives. This makes little sense, given that the temperature increases expected by 2030 are predicted to continue to produce the kinds of extreme weather that we are now regularly exposed to [11]. In this way, the notion of ‘recovery’ is a form of denial, poorly aligned with the reality of those who perceive that they are just waiting for the next extreme-weather-related event to happen.

Lived experience suggests that these combined events are experienced on a continuum, with individuals and communities not having yet ‘recovered’ from one hit before the next hit, and some existing in perpetual anxiety about the next (inevitable) event. This cumulative experience of anticipating and experiencing being under threat leaves individuals and communities with no time for, and a minimal expectation of, ‘recovery’. In addition, this method of characterising events and recovery does not accommodate climate anxiety (variously named, including eco-anxiety, climate grief, etc.), a mental health impact related to perceptions of climate change, coalescing around an existential dread about the future of the planet [12,13] which can be experienced amongst those indirectly as well as directly impacted by extreme-weather-related events [12].

Finally, linked to the previous two points, there is an expectation within the notion of ‘recovery’ that an end point will be reached when individuals and communities have ‘recovered’. This characteristic encapsulates the notion of returning to ‘life as we know it’, which is not always possible or indeed desirable given its lack of attention to inequalities and inequities [14]. Even the more carefully nuanced approaches to recovery still infer an end point. For example, “…people and communities are recovered when they are leading a life they value living, even if it is different to life before the disaster event… [Recovery] is understood as a complex, non-linear, multi-layered process that occurs as people and communities work to resolve the impacts of a disaster” [15] (p. 6, our emphasis). In addition, the expectation of reaching this end point of being recovered is rooted in a deficit model with implied responsibility and accountability placed on those who are ‘not yet recovered’.

## 4. Discussion and Future Directions—A Paradigm Shift Away from Recovery towards Adaptation

We propose an alternative view, which is that not being able to ‘return to life as we know it’ is not a failing but a recognition that this return is not feasible and may not be appropriate. The current assumption of recovery as circular, in which the goal is to return to the starting point with the added wisdom accumulated through this experience, has the potential to set people up to fail. There is a divergence between empirical observation, which shapes the context of mental health risks, and the tantalising but false claim embedded within the current discourse of ‘recovery’ that there can be a safe return to the previous ‘normal’. Encouraging the perpetuation of this imagined goal risks increasing vulnerability to the shock of the inevitable and has the potential to disable more effective pathways for supporting positive mental health and wellbeing.

With the scientific basis for predicting future wide-ranging, cascading (those which lead to other disasters, e.g., floods leading to landslips/landslides [10]) and recurrent disasters and the cumulative impacts of climate change on social-ecological systems, new debates are emerging which highlight the need for disaster planning and recovery models that take into account legacy conditions in recurrent acute disasters [16]; leverage multi-level and multi-sectorial responses for successful rapid adaptation [17]; and include post-disaster strategies that are linked to poverty alleviation, livelihood activities and longer-term development goals [18].

The implications of our observations about ‘recovery’ include its exposed insufficiencies in addressing the needs of individuals and communities enduring the mental health and wellbeing impacts associated with extreme-weather-related events and climate change more broadly and the fact that models (including funding models) for mental health and wellbeing support interventions need to change. For example (as observed in Australia), the model of 10 one-to-one sessions with a psychologist [19] as the only therapy, whilst potentially beneficial on an individual basis, may become less meaningful in this context than an acknowledgement that individuals and whole communities (indeed, ALL of us, as we are all affected by climate change, unlike other exposures) will need input and support in the much longer term. We will also need better platforms for exploring and sharing emotional responses to climate change [20] and robust mechanisms to foster connections with the planet and with others, including the more-than-human.

Notwithstanding the paramount need for radical and sustained mitigation actions [11], we need to shift away from expectations of ‘recovery’ and towards narratives of ongoing adaptation. At the heart of our understanding of adaptation is the notion of dynamic change or transformation. This applies to systems, services and infrastructure, as well as the way in which we live our lives, and embraces the need to relinquish our existing beliefs and expectations of the climate no matter how unnerving this may be [20]. The mental health and wellbeing support system could usefully adopt an anticipatory adaptation approach [2] to planning ahead rather than adapting as a reactive response, with a central focus on building robust and rigorous communities and strengths including informal social connectedness to community, feelings of belonging [21] and purpose. Anticipatory adaptation, for example, can change “…the fundamental attributes of a social-ecological system in anticipation of climate change and its impacts” [2] (p. 7).

There may be resonance here with the concept of ‘recovery’ in the context of chronic illness and, therefore, important lessons to learn about the usefulness and appropriateness of ‘recovery’. Patterns of observations including our perceptions of, and responses to, chronic illness include acceptance of the notion that what supports mental health and wellbeing in the context of chronic illness is a recalibration of both our expectations and understanding of what constitutes successful, positive outcomes. These adaptations can provide new meaning and sense-making pathways and lead to renewed feelings of optimism and hope [20]. Applying this logic to climate-change-related mental health and wellbeing impacts entails a response to the need to adapt to a changed future (in a context where broader systems and structures are often absent) by fostering new pathways of support and care within the community [22] that can increase feelings of belonging and optimism. Higher levels of feelings of belonging and optimism have been shown to be protective of mental health and wellbeing in the context of extreme-weather-related events [21]. This way of thinking points to the uncertainty of the future, not a return to a previous state.

## 5. Conclusions

Whilst there may be a beguiling appeal to the comfort of persisting with this notion of ‘recovery’ from the mental health and wellbeing impacts of climate change, it may undermine our ability to cope by operating as a form of denial. In this way, it is at a minimum disingenuous and at worst actively deleterious to the mental health and wellbeing of those directly and indirectly impacted by extreme-weather-related events and those experiencing climate anxiety. These negative effects include the exhaustion and frustration of those striving and failing to ‘recover’ and a sense of abandonment accompanying the lack of resonance with the lived experiences of extreme-weather-related events and climate change more broadly. These effects can preclude the voices of individuals and communities from being heard. Orientation towards the positive outcomes of ongoing adaptation to sustain mental health and wellbeing may present a more constructive approach to collectively supporting communities’ mental health in response to our changing reality.

## Data Availability

Not applicable.
